# Don't let spurious accusations of pseudoreplication limit our ability to learn from natural experiments (and other messy kinds of ecological monitoring)

**DOI:** 10.1002/ece3.1782

**Published:** 2015-10-26

**Authors:** G. Matt Davies, Alan Gray

**Affiliations:** ^1^ School of Environment and Natural Resources The Ohio State University 412B Kottman Hall 2021, Coffey Road Columbus Ohio 43210; ^2^ NERC Centre for Ecology and Hydrology Bush Estate Penicuik Edinburgh EH26 0QB UK

**Keywords:** Bayesian statistics, confounded effects, hypothesis formation, nesting, peer review, *P*‐values, random effects, scientific publication, statistical population

## Abstract

Pseudoreplication is defined as the use of inferential statistics to test for treatment effects where treatments are not replicated and/or replicates are not statistically independent. It is a genuine but controversial issue in ecology particularly in the case of costly landscape‐scale manipulations, behavioral studies where ethics or other concerns may limit sample sizes, ad hoc monitoring data, and the analysis of natural experiments where chance events occur at a single site. Here key publications on the topic are reviewed to illustrate the debate that exists about the conceptual validity of pseudoreplication. A survey of ecologists and case studies of experimental design and publication issues are used to explore the extent of the problem, ecologists’ solutions, reviewers’ attitudes, and the fate of submitted manuscripts. Scientists working across a range of ecological disciplines regularly come across the problem of pseudoreplication and build solutions into their designs and analyses. These include carefully defining hypotheses and the population of interest, acknowledging the limits of statistical inference and using statistical approaches including nesting and random effects. Many ecologists face considerable challenges getting their work published if accusations of pseudoreplication are made – even if the problem has been dealt with. Many reviewers reject papers for pseudoreplication, and this occurs more often if they haven't experienced the issue themselves. The concept of pseudoreplication is being applied too dogmatically and often leads to rejection during review. There is insufficient consideration of the associated philosophical issues and potential statistical solutions. By stopping the publication of ecological studies, reviewers are slowing the pace of ecological research and limiting the scope of management case studies, natural events studies, and valuable data available to form evidence‐based solutions. Recommendations for fair and consistent treatment of pseudoreplication during writing and review are given for authors, reviewers, and editors.

## Introduction: What is Pseudoreplication Anyway?

Most ecologists can probably vividly remember when they first became aware of the issue of pseudoreplication. In our case, the problem was carefully explained by our PhD supervisor after we began to consider the minefield of experimental design. This also involved us sitting down, with some trepidation, to read the seminal paper by Hurlbert (1984). Later, and particularly when we were given papers to discuss in class, we became rather proud of our ability to spot the issue and probably took far too much pleasure in pointing it out to other students and colleagues. At least one of us was formerly rather too keen on pointing it out during peer review. Let us be clear at the outset, there is no doubt that Hurlbert did the science of ecology a very significant service by drawing attention to the limits of statistical inference imposed by poor experimental designs. The importance of his work is easily demonstrated by the all‐seeing eye of Google, which indicates it has been cited nearly six and a half thousand times in the 30 years since. Hurlbert (1984) actually described four different types of pseudoreplication but, in our experience, “simple pseudoreplication” and “temporal pseudoreplication” are probably the most common. Simple pseudoreplication can arise when researchers define a hypothesis and collect what they deem to be independent samples from two populations they wish to compare, when in reality the samples are not independent because, for example, each sample is collected from a single monitoring unit. Temporal pseudoreplication occurs when differences in the timing of data collection between experimental units mean that time and treatment effects are confounded or when repeated measures designs are treated inappropriately.

The above forms of pseudoreplication remain genuine problems that, where designed experiments are concerned, often have no excuse. The recent reviews by Waller et al. (2013) and Ramage et al. (2013) highlight the potential dangers associated with pseudoreplicated experiments. Waller et al. (2013) found that 39% of primate communication studies were pseudoreplicated, but in 88% of these, the issue was avoidable. Ramage et al. (2013) found that 68% of studies on the effects of logging on the biodiversity of tropical rainforests were pseudoreplicated and, by comparing species composition in a number of contiguous forest research plots, demonstrated that rates of false inference of “treatment” effects can be between 0% (tree composition) and 69% (stingless bee composition) depending on the taxon in question. While this is certainly a cause for concern, unfortunately, as Ramage et al. (2013) and Waller et al. (2013) admit, designed experiments aren't always possible or desirable. If we adopt a militant stance to pseudoreplication, opportunities could be lost to learn from disturbance events, “natural experiments,” and large/landscape‐scale manipulations where ecologists arrive after the fact or replication is simply not feasible. Such studies face the issue that, although most ecologists are aware of the basic principle of pseudoreplication, many either have difficulty understanding it, appear reluctant to consider the practicalities involved, and most importantly may be unaware of its potential solutions. We know this because for a long time, we could be classified into more than one of those categories. We're fairly sure respondents to this article will argue pretty convincingly that we still can be!

Through personal experience and communication with colleagues, we began to realize that there's a problem when editors and reviewers citing pseudoreplication, fairly or not, become the kiss of death for papers. By drawing attention to the issues associated with pseudoreplication, we won't claim to be particularly original as previous authors (e.g., Hargrove and Pickering 1992; Oksanen 2001; Cottenie and De Meester 2003; Feeberg and Lucas 2009; Schank and Koehnle 2009) have all discussed, critiqued, and argued over the concept in some detail and made a number of their own recommendations (Table 1). However, it alarms us that consideration of the reality of ecological research still seems to be lacking during the review process and that reviewers and editors are rejecting papers while quite clearly not understanding the nuances of the issue. This exacerbates the “file drawer problem” (Rosenthal 1979) and thus prevents useful data being published that would otherwise increase the scientific evidence base upon which solutions and adaptations to environmental change can be formed.

**Table 1:** Previous authors’ solutions to the problem of pseudoreplication and potential issues and pitfalls. Many of these suggestions will match our own, and the issue is therefore much more a function of some researchers’ and editors’ attitudes and perceptions of the issue

Proposed solution	References	Issues
Authors should clearly articulate potential confounding effects. Be explicit about experimental designs	Schank and Koehnle (2009), Oksanen (2001)	Gives ammunition to reviewers who often seem to dislike studies without “perfect” designs
Compare a single treatment with multiple controls	Oksanen (2001)	Many statistical tests require variance estimates for treatment and control
Where site and treatment are confounded, examine magnitudes of difference between treatment and control areas before and after the experiment	Oksanen (2001)	Requires information on predisturbance conditions
Utilize meta‐analysis to investigate cross‐study comparisons	Hargrove and Pickering (1992), Cottenie and De Meester (2003)	File drawer problem and bias against pseudoreplication means many observational studies and negative results are not published
Accompany presentation of all results with inferential statistics	Oksanen (2001)	Care needed to avoid over interpretation if, for example, sites and treatments are confounded
Use inferential statistics to assess the “reliability” of descriptive statistics	Cottenie and De Meester (2003)	Care needed to avoid over interpretation
Focus on effect sizes, “how different the two statistical populations must be,” and divergence/convergence of temporal trends	Oksanen (2001)	Editors and reviewers (still) routinely demand *P*‐values
Avoid pooling of observations and instead use multilevel modeling as a statistical solution.	Waller et al. (2013), Schank and Koehnle (2009)	More complex statistical methods needed which require expertise
Incorporate turnover‐by‐distance relationships and environmental data into their analyses to assess potential for spurious detection of significant differences.	Ramage et al. (2013)	Complex statistical analyses needed
Utilize Bayesian statistics	Oksanen (2001)	You'll need to understand Bayesian statistics first!
Carefully consider and clearly state the statistical inferences that can be drawn from data sets	Ramage et al. (2013), Schank and Koehnle (2009), Oksanen (2001)	Provides ammunition to those reviewers and editors looking for a reason to reject papers. Traditional journals like to maintain high rejection rates.
Pseudoreplication often related to confounded effects that require careful interpretation	Schank and Koehnle (2009)	See above
Explicitly state the limited scope of the results	Cottenie and De Meester (2003)	No one seems to want to publish “case studies”
Permit use of “normic statements” that hypothesize about what would normally occur given the results from a particular case study or statistical test	Hargrove and Pickering (1992)	Reviewers seem to dislike speculation. It would be better to phrase normic statements as new hypotheses to test
Substitute statistical inference for ecological inference	Hargrove and Pickering (1992)	Requires acknowledgment of precisely what statistical tests are comparing (e.g., site vs. treatment differences)
Allow publication of studies without inferential statistics	Hurlbert (1984)	Prevents authors from examining the extent to which observed differences are meaningful. Editors and reviewers (still) routinely demand *P*‐values
Avoid use of term pseudoreplication during review and instead specifically describe perceived statistical problems	Oksanen (2001)	No argument from us here!
Do not automatically reject “experiments” where there is no treatment replication	Hurlbert (2004)	We couldn't agree more!
Pseudoreplication should be taken into account when applicable	Cottenie and De Meester (2003)	Allows continued use of an imprecise term and doesn't encourage reviewers to specify exact statistical issues

## Methods and Results: Is it Just us?

We were keen to ensure that it wasn't just us getting frustrated by the peer review process or, even more worryingly, trying to pass off poor quality research that could have been designed better! We therefore used a survey of ecologists to gain an idea of the extent to which other researchers encountered the problem and faced issues when trying to publish.

The online survey was disseminated through our professional network and advertised on the Ecolog‐l mailing list and Twitter. There was much interest in the topic, and 103 responses were collected which revealed the following key findings:

1. Fifty‐eight percent of respondents had faced a research question where they felt pseudoreplication was an unavoidable issue (Table 2).
  **Table 2:** Categories of pseudoreplication problem identified in the questionnaire and the frequency with which respondents described them

Landscape‐scale treatments/monitoring (including manipulations of forest stand structure)	10
Nested designs with insufficient replication at site level	9
Wildlife behavior/physiology (including repeated measures on a small number of individuals)	9
Confounded site/stand and treatment (including multisite vegetation chronosequences)	8
Demography and disease and – what is the appropriate analysis level site, plot, or individual?	8
Exclosures at a single site (including grazing and irrigation studies)	6
Aquatic ecology + hydrology ‐ unreplicated ponds/lakes/watersheds	5
Fire behavior and effects (including studies of individual wildfires)	5
Single‐site case studies or phenomena limited to one location	5
Spatial autocorrelation	3
Repeated measures of vegetation change (including studies on a single relevé)	2
2. Of those who'd faced the problem, 85% were aware of the concept before they started their research although most (89%) were not discouraged by it.
3. Nearly 70% of respondents had read Hurlbert (1984).
4. Two‐thirds of respondents tried to deal with the issue during their statistical analysis or by acknowledging the limits of statistical inference possible given their design. A third dealt with the issue during hypothesis formation by clearly defining their population, and a fifth framed their conclusions as new hypotheses. Only four respondents admitted they just hoped no one would notice (which is honest but naughty!).
5. Half of the respondents admitted they'd had difficulties getting their research published, and 17% were never able to get their studies published at all. Of those who experienced publication difficulties, 41% received major corrections but 55% had their paper rejected (with less than half of those being given the option to resubmit). A quarter of the respondents had ended up in prolonged arguments with reviewers and/or editors.
6. When completing peer reviews, reviewers who had not encountered pseudoreplication issues in their own research, though a relatively small proportion of all reviewers (25%), appeared to be considerably more likely to reject or ask for resubmission of papers with pseudoreplication (59%) than those who'd had to deal with the issue themselves (36%).

The survey revealed that we aren't the only ecologists who are frustrated by their experiences during peer review, and it was clear a number of respondents had particularly strong views (Box 1). All but one of the respondents who provided comments expressed frustration with the way pseudoreplication was dealt with during review. The sample of respondents was probably self‐selecting (Olsen 2008), but it does indicate that there is a proportion of scientists genuinely concerned about the issue. This is also evidenced by the continuing and ongoing debate (Hargrove and Pickering 1992; Oksanen 2001; Cottenie and De Meester 2003; Feeberg and Lucas 2009; Schank and Koehnle 2009; Ramage et al. 2013). Most views expressed in the survey could be categorized as feeling that:

Box 1 **Selected quotes illustrating the dominant themes that emerged from the online pseudoreplication questionnaire.**

**Table:** 

Resource issues	Statistical solutions
“The issue was always resulting from the balance between what is	“They [the reviewers] were too focused on the possibility and
reasonably possible and what is ideal… People scream	effects of pseudoreplication than our approach to dealing with it”
pseudoreplication when it's not pseudoreplication”	
	“Hurlburt did us all a disservice when he said that statistics can't
“…pseudoreplication is an issue that's been blown way out of	be used when pseudoreplication is present. They can but what
proportion. The real issue is how you interpret your results and	they tell you is something that warrants careful interpretation.”
then report them. How do you replicate things like marshes, forest	
patches? You have to say, what I found is true for this forest and	“Pseudoreplication is a bogus term for a poorly nested design. The
then the question is how representative that forest is of all the forests in the area”	Hurlbert publication is one of the most pernicious publications in all of ecology.”
“Ultimately all field work is pseudoreplicated, depending upon	“Pseudoreplication is just a question of correct model
scale. I have been criticized because all my work occurred in 1	specification. If the model correctly reflects the sampling design,
estuary, 1 only in the Gulf of Mexico”	then the issue becomes one of parameter estimation and
	potential parameter nonidentifiability.”
“Often there is simply not the funding to conduct landscape scale	
experiments without pseudoreplication”	“I would say “generalized linear multilevel models” but, yes,
	basically random effects”
“Replication is not always possible in ecology. This is particularly	
true in restoration ecology when restored ecosystems are created	“I used multiple control sites, to at least differentiate the
at great expense and cannot always be replicated for the purpose	treatment area from multiple other sites.”
of scientific study. Sometimes we just have to study what is	
there!”	“Indicating that samples were taken at a distance greater than the
	autocorrelation distance for many soil variables (from the
“In many ways, it is very difficult to really meet the needs of	literature in the same ecosystem) and framing the results and
replication and even when we do it is often somewhat arbitrary. In	conclusions to this experiment design.”
many instances, the research we have done could be better	
referred to as ‘case studies,’ but then we'd have to pray a journal	
will accept that.”	
“Ecologists are so hung up on pseudoreplication, it's not even an	
issue for my hydrologist colleagues, in whose research	
pseudoreplication is often inevitable.”

1. pseudoreplication was inevitable in many types of research due to cost, scale, and other “real‐world” environmental issues such as a wildfire, drought, or flood only occurring once;
2. many kinds of pseudoreplication can be dealt with statistically using appropriate nesting or random effects.

## Discussion

The two broad sets of issues identified above provide important guidance for reviewers and editors that have to consider papers where pseudoreplication arises. We consider that there are three key questions that should be asked in such a situation and which it's useful to consider in a little more detail and with a couple of specific examples from our own experience:

### Question 1: Has potential pseudoreplication been accounted for in the formation of hypotheses and subsequent interpretation?

The issue of simple pseudoreplication is very frequent where researchers are trying to understand the effects of unusual individual events such as a flood (Friedman et al. 1996), windstorm (Nagel et al. 2006), wildfire (e.g., Maltby et al. 1990; Johnstone and Kasischke 2005), or volcanic eruption (del Moral and Lacher 2005). The survey revealed that the problem also frequently arises in aquatic ecology and hydrology, where it is difficult to get adequate replication of different ponds, lakes, or watersheds, and in studies involving landscape‐scale effects such as manipulations of forest structure, and the study of fire and grazing effects. Overzealous application of the pseudoreplication concept would have it that it is impossible to test hypotheses about the effects of individual events or where site and treatment are confounded. Strictly this is correct as with, for example, a single disturbance and with monitoring plots located on either side of the disturbance's boundary, one cannot statistically separate spatial effects from “treatment” effects. Potentially, this is a genuine issue as demonstrated by the existence of spatial autocorrelation – put simply that plots closer to one another will tend to be more similar than those further away (Legendre 1993). In fire ecology, it's a particular issue as wildfire perimeters can occur where changes in fuel structure and moisture lead to fires self‐extinguishing. This of course means that burnt and unburnt areas are not comparable. More widely, effects may in part be caused by confounding underlying variables such as variation in soil type, soil moisture, aspect, or elevation. We think, however, that the issue of confounded effects should be firmly separated from the issue of pseudoreplication – often they seem to be used interchangeably, but one does not necessarily imply the other.

A paper recently submitted by the one of the authors here, and describing the effects of stock removal on landscape‐scale patterns in species richness (Davies and Bodart 2015), encountered just such a problem. The monitoring described in the paper examined differences in vegetation on either side of a single fenced exclosure used to remove stock from roughly half of a 640‐ha farm in the Scottish Southern Uplands. We tested for differences in species richness and community composition on either side of the fence using a linear model and PERMANOVA (Anderson 2001), respectively. The paper was rejected from one journal and delayed in a second due to accusations of pseudoreplication. However, Hurlbert does not state that it is impermissible to test for differences in cases like the example described above (Hurlbert 1984; Kozlov and Hurlbert 2006). He did explain that on the basis of such a test, one cannot infer statistically that the treatment caused any observed effect. In our case, we specifically acknowledged the problem and stated at the outset that we were testing for differences between either side of the fence at our site (rather than for the effect of grazing per se). Our plots were independent samples of vegetation on either side of the fence at our site. We made no statistical claims about the effect of grazing removal but did interpret the results in light of our ecological understanding of the system and its management. We used our results to form new hypotheses about the effect of grazing removal which we acknowledged would require a wider study to validate. We ended up probably being overcautious as there really was no other reasonable explanation for the differences we saw.

Hurlbert seems to have made it clear that such an approach is permissible (Kozlov and Hurlbert 2006) even if it is frustrating and might, to some, seem a little like cheating. However, our paper drew a clear line between what we can *infer* as statisticians and what we can *interpret* as ecologists. Reviewers should examine the design of natural experiments carefully to decide whether it is likely that confounding variables are driving any spatial differences. In our case, we took pains to ensure our grazed and ungrazed plots were close together, located on the same soil type, at the same elevation, and on the same aspect. Reviewers should feel at liberty to ask authors to acknowledge the potential issue, to describe any mitigating action they took in their methods, to be cautious in their interpretations, and to phrase conclusions as new hypotheses. This is supported by Oksanen (2001) who stated that “it is reasonable to require that the author is explicit about his/her epistemological position and about the design of the experiment.” It is not reasonable for reviewers and editors to just chuck out studies on the basis of on‐the‐ground realities that impose limitations on experimental designs. Extreme or unusual events are, by definition, rare and unreplicated, and ecologists should not shy away from learning from them. In fact, we should probably be encouraging more studies on them to build our insufficient evidence base. A choice example from the survey was a respondent who studied a particular form of a wildlife behavior that only occurred on a single island and which they compared with that on other islands – the paper was rejected as pseudoreplicated as there was only a single atypical island! Presumably, individuals forming the population of each island were used as the sampling unit but that was appropriate in this case. This use of a single “treatment” compared against several “controls” also matches one of Oksanen's (2001) recommendations.

### Question 2: Is it reasonable to wonder whether a fully replicated experimental or manipulative study is an alternative?

**Figure 1:**
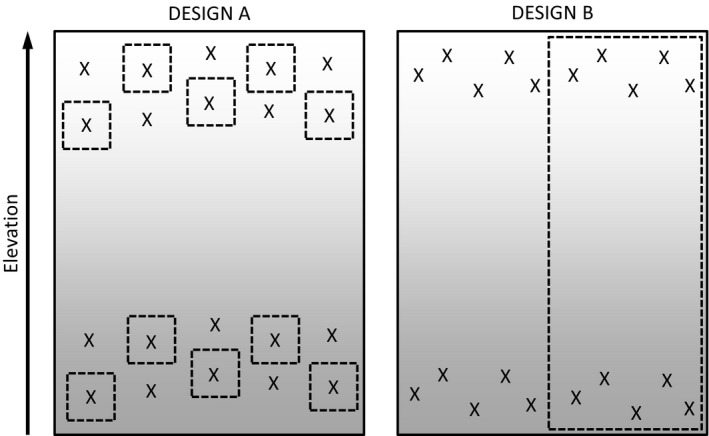
Hypothetical example of two experimental designs examining the effects of some form of disturbance (e.g., wildfire, grazing) on vegetation structure at two different elevations. Design A is a formal experiment, whereas Design B is a researcher's response to a natural (i.e., unplanned by the researcher) event. Dotted lines mark the perimeter of the disturbances which could be, for example, a series of experimental fires in A and a wildfire in B or a number of grazing exclosures in A vs. a landscape‐scale fence in B. Assuming that soils, slope, and aspect are more or less homogenous, at least within each studied elevational band, are the results of Design A more ecologically meaningful than those in B? Which design more adequately captures the ecological reality of wildfire or landscape‐scale alterations to grazing management? We would argue that Design A might actually sacrifice ecological reality for statistical independence as, for example, small fires cannot mimic a wildfire event and small grazing exclosures do not allow natural movement of grazers across landscapes.

In cases like the lead author's grazing research (Davies and Bodart 2015), one should also consider whether, had this been a strict “experiment”, it would have been found acceptable. In the case of the above study, had we established an experiment at our site with multiple small experimental grazing exclosures (Fig. 1) and monitored changes in them compared to outside, we suspect we would not have been criticized even though, ecologically, the results would have been no more meaningful than the natural experiment we were faced with. The multiexclosure design would, in that case, have been seen as a “real experiment,” and we suspect most experiments are completed at single sites (though ideally they wouldn't be). Ecologically, we find it hard to see how our results would be different or more valid in this situation; in fact, edge effects in the small “independent” exclosures could have been an issue. If a reviewer examining Figure 1 feels they could accept Design A, then, with the appropriate caveats, they should not reject Design B. This also makes the point that what we define as our statistical population may often be somewhat arbitrary anyway as boundaries between geographical regions and ecological communities are rarely entirely solid. In the study of interactions between grazing and elevation what population does our sampling need to represent – grasslands at our site, *Festuca – Molinia* grasslands in southwest Scotland, upland grasslands in the UK, all grasslands in Europe, or all grasslands in the world ever? Of course what is appropriate all depends on the nature of their inferences we want to make – Are they about grasslands in general or limited to our site?

Case studies, where pseudoreplication may be an issue, have been criticized by some authors. Miller et al. (2003), for example, point to the fact that 42 of 56 studies of forest roosting bats they reviewed were “impact assessments” providing a site‐ and case‐specific investigation of a potential impact with no replication or randomization and inferences spatially limited to the site and impossible for treatment effects. We'd agree with them that it would be nice to have more manipulative experiments of the effect of forest structure, but there's a reason why they couldn't find any in the literature, they're costly, difficult to implement, hard to find adequate sites for, and impossible to maintain. We can see their concerns that the “speculative and hypothetical nature of explanations for causality based on observational studies often is lost through time and becomes dogma” but equally would argue that it'd be silly to state that we learned nothing useful from those 42 case studies. In addition, the increasing use of Bayesian approaches to the analysis of ecological data allows incorporation of prior information that can be collated from previously published data. The use of Bayesian methods and presenting prior information also makes study results more useful for meta‐analysis, another technique that has increased in the last few decades (Gurevitch et al. 2001; Koricheva et al. 2013). The use of these approaches suggests that case study research can be of considerable value.

Unfortunately, site‐specific case studies seem to be of declining interest for many journals highly concerned with maintaining IMPACT factors. For us and many managers, conservationists, and policymakers, the frustrations in trying to get lessons learned from monitoring studies out of the file drawer and published, so we can facilitate evidence‐based environmental management, is a real concern. How much is science missing? We have at least one study where, after three years of trying, it was assigned to the file drawer because it was repeatedly rejected due to its spatially and temporally pseudoreplicated design. Did the study have no scientific merit? Well the reviewers obviously thought so, but this was one of the first studies to report carbon dioxide and methane emission from peatlands in relation to disturbance from grazing and fire, an area of ecology that we are still largely clueless on.

### Question 3: Has potential pseudoreplication been accounted for in the statistical analysis?

Correctly describing an experiment as pseudoreplicated requires that reviewers have a solid understanding of an experiment's design and the statistical treatment of the design by the authors. Often, this may not be the case either because reviewers are unfamiliar with the ever‐increasing array of powerful statistical tests available, or more likely because the authors haven't explained their design and analysis clearly enough the first time round. A good example comes from some long‐term but ad hoc monitoring of variation in the fuel moisture content of *Calluna vulgaris* (L.) Hull (Davies et al. 2010). The data were collected from multiple sites over a number of different years and seasons as time, labour, and funds were available. A key issue in the final data set was that site was confounded with year and season as data from some sites were only available for a single season. Within each season at each site, we collected data over a number of different days, and from a number of different quadrats and subquadrats. In our analysis, we were interested in the proportion of variance explained by each of the different levels of our hierarchical “experimental” design Site/Season – Day – Quadrat – Subquadrat. Publication was delayed by more than 18 months as we were accused of pseudoreplication due to the confounded site/season effect. We were rejected from our first journal, and in the second, only the intervention of a statistician, sensibly brought in by the editor, ended the ding‐dong between us and one of the reviewers. The accusation of pseudoreplication was incorrect as in our analysis, we used a random effects model where Day (and thus all levels below that) was nested within Site/Season. At no point did we claim there was a significant difference between sites or seasons or state that we were testing that hypothesis; a benefit/frustration of lmer in the lme4 package (Bates et al. 2014) of R (R Core Team 2014) is that it doesn't provide *P*‐values anyway! This makes the first important point – useful ecological data often come from messy monitoring, designs that might appear pseudoreplicated but which can be dealt with using appropriate statistical techniques. The current debate on the value of *P*‐values in ecology, illustrated by a recent special issue of Ecology (Ellison et al. 2014), is particularly pertinent when the data are messy or from a single site; we do not think we are alone in having been asked by reviewers to provide *P*‐values when they are simply not appropriate or informative. These ongoing debates and the development of new techniques all point to the fact the ecological statistics palette is ever‐increasing and many new approaches make the concept of pseudoreplication irrelevant.

As a number of authors have pointed out (e.g., Feeberg and Lucas 2009), Hulbert's original paper (Hurlbert 1984) was written more than 30 years ago and increased computing power means there are a number of analytical options for dealing with pseudoreplication. The use of random effects and nested model designs can be important tools in this regard, but using such approaches will not be possible if there are only single treated and untreated sites. Our survey suggests that many researchers do use mixed‐effect modeling approaches to deal with pseudoreplication problems including studies involving repeated measures on sites or individuals. In most cases, what we would attempt to do with these approaches is to account for, or “partial out,” the uninteresting or troublesome variance associated with, for example, the site effect, and estimate the response and treatment variance associated with our hypotheses. However, this may not always be appropriate; Oksanen (2001), for example, cites examples along productivity gradients where he considers using site as a random factor, but concludes this “may look clean, but the statistics then focus on the question whether there are some unspecified spatial differences in the ecological processes to be studied.” This emphasizes the need for clearly stated hypotheses and statistical methods, and that both authors and reviewers have presented and interpreted the statistics correctly.

In summary, we would argue an experimental design is only pseudoreplicated within the context of the statistical tests that are applied to it, the hypotheses that are being tested and the conclusions that are drawn from that analysis. Statistical solutions may not always be available, and in such cases, a clear definition of what is being inferred from the tests is required (see Question 1).

## In Conclusion

One of the most concerning things about the survey was that respondents' attitudes when reviewing seemed to be strongly influenced by their ecological background – those who'd encountered the issue of pseudoreplication in their own work were much less likely to reject papers. This appears to confirm what some respondents suggested, that there's a split in ecology between stricter experimentalists and those focused on more applied problems. This lack of a consistent approach is a problem. Oksanen (2001) went as far as to state “The term pseudoreplication has been so much abused that its value in a review is questionable. Referees should preferentially refrain from using it” and that “to require that inferential statistics should not be used in the context of unreplicated experiments is plain nonsense.” Whatever your own opinions, reviewers need to realize that the debate about pseudoreplication is ongoing and not at all settled. Although we don't really want to end up in the kind of philosophical battle seen between Hurlbert (2004) and Oksanen (2004), we argue that accusations of pseudoreplication should not be made without appropriate effort to demonstrate the accusation is statistically valid and ecologically meaningful. Pseudoreplication should not be the death knell it has become for scientific papers. Messy ecological data are a fact of life when monitoring has to be completed as and when possible, in the context of the high costs of fieldwork and where researchers seek to utilize historical data and natural events. Natural experiments are a vital component of ecological research and should not be subject to a game of pseudoreplication Russian roulette when submitted. Pseudoreplication is not an inherent feature of an experimental design but rather occurs within the context of the hypotheses that are formed alongside the experimental design and in the statistical treatment of data. As Hurlbert (2004) points out, there are a variety of statistical approaches to deal with the issue, but it can also be mitigated by appropriately framed hypotheses, an appreciation of the limits of statistical inference and appropriate caution in the interpretation of the results of statistical analyses. Failing that perhaps more authors, reviewers, and editors should be willing to take Hurlbert's (1984) advice: “Be liberal in accepting good papers that refrain from using inferential statistics when these cannot validly be applied.” This is important as meta‐analysis (Harrison 2011) becomes an ever more influential means by which individual studies can be combined and analyzed to detect consistent patterns. We would suggest that this is particularly important for natural experiments where site and disturbance/treatment effects may be confounded (e.g., Ramage et al. 2013). In order to allow meta‐analyses (or comprehensive nonstatistical reviews), we first have to allow the publication of case studies and experiments on which the process depends (including negative results). Previous authors have made many sensible recommendations nearly all of which we agree with, although we have noted a few caveats (Table 1). Here we specifically recommend:

### Authors

1. When designing monitoring campaigns, consider the use of multiple control sites/plots to account for spatial variability in study systems but don't be put off if this is just not possible.
2. Be honest and up‐front about pseudoreplication and take a couple of lines to explain how you've dealt with it in the formation of your hypotheses and/or in the specification of your statistical models. Be clear about how you define the population in the context of your study.
3. Get statistical advice on whether approaches like nesting and random/mixed‐effects modeling could help you deal with your problem – resist the comfortable dogmatic statistical approach (e.g., ANOVAs and *P*‐values); there's often better alternatives.
4. Be cautious about the extent to which you ascribe causation to pseudoreplicated treatment effects. Frame your conclusions as new hypotheses to test – Isn't that how science is meant to work anyway?
5. The onus is on you to clearly justify the level of your experiment you define as a sample, to show that pseudoreplication is unlikely to be associated with confounded effects and/or that you've accounted for such issues in your analysis.

### Reviewers

1. Put yourself in the place of the authors – is there any reasonable way in which the experiment could have been designed differently given realistic time/funding constraints.
2. Back up your accusations and ensure you understand authors’ experimental designs and statistics before you select “reject.” If you believe pseudoreplication is an issue, you should explain in detail why, and why the authors’ explicit or implicit hypotheses and analyses are inappropriate in the context of the population of interest. Is there an alternative statistical technique you could suggest if the analysis is inappropriate?
3. If you really believe effects could be confounded, provide a realistic argument to show why, for example, it's ecologically less likely that treatment effects drive differences than site effects.
4. If you have concerns, don't just reject the paper outright; rather, give the authors a chance to explain their approach and, if necessary, ask that the editor gets the opinion of a statistician. It's more than likely the authors are aware of the problem and have attempted to deal with it but haven't explained this clearly enough.

### Editors

1. We are moving into a new era where page space is becoming a meaningless concept, so there are opportunities to be more sparing with rejection. We know you get more submissions than you can publish but make sure you're serving science and the wider community by being as cautious with rejections as you are with acceptances.
2. Have a reliable and knowledgeable statistician to call on when accusations of pseudoreplication arise and there's argument over the statistical treatment of data.
3. Finally, don't blindly accept accusations of pseudoreplication but ask reviewers to back up their accusation in detail.
